# Distinct frontal and amygdala correlates of change detection for facial identity and expression

**DOI:** 10.1093/scan/nsv104

**Published:** 2015-08-04

**Authors:** Amal Achaibou, Eva Loth, Sonia J. Bishop

**Affiliations:** ^1^Department of Psychology and Helen Wills Neuroscience Institute, UC Berkeley, CA 94720, USA and; ^2^Sackler Institute for Translational Neurodevelopment, Institute of Psychiatry, Psychology and Neuroscience, King’s College London, London SE5 8AF, UK

**Keywords:** amygdala, change detection, expression, faces, identity, prefrontal

## Abstract

Recruitment of ‘top-down’ frontal attentional mechanisms is held to support detection of changes in task-relevant stimuli. Fluctuations in intrinsic frontal activity have been shown to impact task performance more generally. Meanwhile, the amygdala has been implicated in ‘bottom-up’ attentional capture by threat. Here, 22 adult human participants took part in a functional magnetic resonance change detection study aimed at investigating the correlates of successful (*vs* failed) detection of changes in facial identity *vs* expression. For identity changes, we expected prefrontal recruitment to differentiate ‘hit’ from ‘miss’ trials, in line with previous reports. Meanwhile, we postulated that a different mechanism would support detection of emotionally salient changes. Specifically, elevated amygdala activation was predicted to be associated with successful detection of threat-related changes in expression, over-riding the influence of fluctuations in top-down attention. Our findings revealed that fusiform activity tracked change detection across conditions. Ventrolateral prefrontal cortical activity was uniquely linked to detection of changes in identity not expression, and amygdala activity to detection of changes from neutral to fearful expressions. These results are consistent with distinct mechanisms supporting detection of changes in face identity *vs* expression, the former potentially reflecting top-down attention, the latter bottom-up attentional capture by stimulus emotional salience.

## Introduction

The ability to rapidly detect changes in our environment is important for survival. However, even large changes can pass unnoticed. This has been the subject of much research and has led to recognition of phenomena now known as ‘inattentional blindness’ (where focused attention on one element of a scene can lead to another element being missed altogether) and ‘change blindness’ (where even in the absence of attention being overtly directed elsewhere a change in a scene or object can be missed if a brief visual disruption occurs; Rensink *et al.*, 1997). The role of top-down attention is well recognized in inattentional blindness, and may also influence performance on change detection tasks. In relation to the latter, it has been demonstrated that engagement of frontal regions implicated in top-down attentional control prior to the change itself is higher on trials where change detection is successful than on trials where changes are missed (Pourtois *et al.*, 2006). This might well reflect trial to trial fluctuations in alertness and allocation of attention to the task at hand leading to differences in change detection performance. Consistent with this proposal, pre-trial fluctuations in ‘instrinsic’ activity in frontal regions has indeed been shown to covary with performance on attentional tasks (Coste *et al.*, 2011; Nozawa *et al.*, 2014).

Attention to the task at hand—e.g. monitoring for changes in certain features of the stimuli viewed—may not, however, be the only determinant of change detection. To quote Most and colleagues, ‘From a standpoint most applicable to everyday life, the question of why people fail to notice unexpected items can be inverted, rephrased to inquire, “What kinds of stimulus properties and/or perceiver-controlled processes influence the likelihood that someone will notice an unexpected object or event?” (i.e. What will capture awareness […])’, (Most *et al.*, 2005). While momentary changes in task-focused attention may be an example of a pertinent perceiver-based process, a strong contender for pertinent stimulus property is stimulus emotional salience, and in particular whether a change indicates the potential introduction of threat into the environment. In line with this, there is some behavioral evidence that changes involving the introduction of a threat-related stimulus into a visual scene are more likely to be detected (Mayer *et al.*, 2006; Lyyra *et al.*, 2014). Meanwhile, neuroimaging findings suggest amygdala activation to emotionally salient, especially threat-relevant, stimuli can lead to the ‘bottom-up’ capture of attention (Vuilleumier and Driver, 2007). In the context of change detection, such amygdala-driven ‘bottom-up’ attentional capture might potentially facilitate the detection of emotionally salient changes, overriding the impact of fluctuations in top-down attention.

Here, we address whether successful detection of emotionally salient *vs* non-emotionally salient changes does indeed differentially rely on amygdaloid *vs* frontal circuitry using a class of stimuli, faces, where the ability to detect both emotional and non emotional changes is of pertinence to everyday social interaction. We adapted a change detection task used previously (Beck *et al.*, 2001) to incorporate changes in facial expression as well as facial identity. Participants saw two consecutive displays of pairs of images separated by a short gap and had to report if they detected a change in either image while functional magnetic resonance (fMRI) data were acquired. We tested three hypotheses. First, that fusiform cortex would show an increased response for detected *vs* undetected changes for both changes in identity and expression, in line with activity in this region tracking perceived differences in facial stimuli (Fox *et al.*, 2009; Xu and Biederman, 2010). Second, that detected *vs* undetected changes in facial identity would be associated with increased prefrontal activity, in line with trial to trial variations in engagement of ‘top-down’ attentional mechanisms influencing detection of relatively low salience identity changes. In contrast, detected *vs* undetected changes in facial expression (from neutral to fearful) were not expected to be differentiated by the extent of prefrontal activity, in line with bottom-up capture of attention by stimulus emotional salience, in particular threat-relevance, overriding the influence of fluctuations in top-down attention upon successful change detection. Following on from this, our third hypothesis was that successful detection of neutral to fear expression changes would instead be associated with increased amygdala activity.

## Materials and methods

### Participants

Twenty-four participants (16 males, 8 females, mean age ± SD: 27.5 ± 8 years) completed a change detection task while fMRI data were acquired. The study was approved by the Local Research Ethics Committee and carried out in compliance with their guidelines. Written informed consent was obtained from all participants prior to participation. Individuals with a history of psychiatric care, neurological disease or head injury were excluded from the study, as were individuals using psychotropic drugs or with a significant history of illegal drug use. Participants who were outliers for performance in any condition were excluded from further analysis. Two participants had very low performance for the house no-change condition (% responses correct being 50 and 53% compared to a mean 91 ± 0.9, range 70–100% for the remaining participants) and were therefore excluded. This left data from 22 participants (14 males, aged 19–49 years, mean age ± SD = 27.5 ± 8 years).

### Stimuli

Stimuli were grey-scale images of faces and houses. The face stimuli used comprised eight different individuals (4 males, 4 females) taken from the Pictures of Facial Affect (Ekman and Friesen, 1976), displaying neutral expressions or different intensities of fearful expressions. The faces were cropped to remove non-face information (e.g. hair) and outer face contours. The house stimuli comprised eight different greyscale images of houses taken from a previously used set (Bishop *et al.*, 2004a,b). We manipulated difficulty of change detection by using Fantamorph software (Abrosoft Inc.) to create face morphs part-way between neutral and fearful expressions and part-way between different identities. Our final stimuli included fully neutral expressions, low fear (40% fearful/60% neutral) and high fear (60% fearful/40% neutral) expressions. In addition new morphed identities were created from faces of the same gender, these comprised 20% of one given identity and 80% of a second identity. Our key conditions of interest were ‘small neutral to fear’ changes where a neutral face was replaced by a low fear face for the same identity, ‘large neutral to fear’ changes, where a neutral face was replaced by a high fear face for the same identity, ‘small identity’ changes, where a neutral face of one identity was replaced by a neutral face morphed 80% towards a second identity, and ‘large identity’ changes, where a neutral face of one identity was replaced by a neutral face of a second identity. Pilot data confirmed that participants performed above chance in all conditions with better performance for ‘large’ than for ‘small’ changes, *F*(1,12) = 59.844, *P* < 0.001, and no significant effect of change type or interaction between change type and change size.

### Experimental procedure

**Fig. 1.**
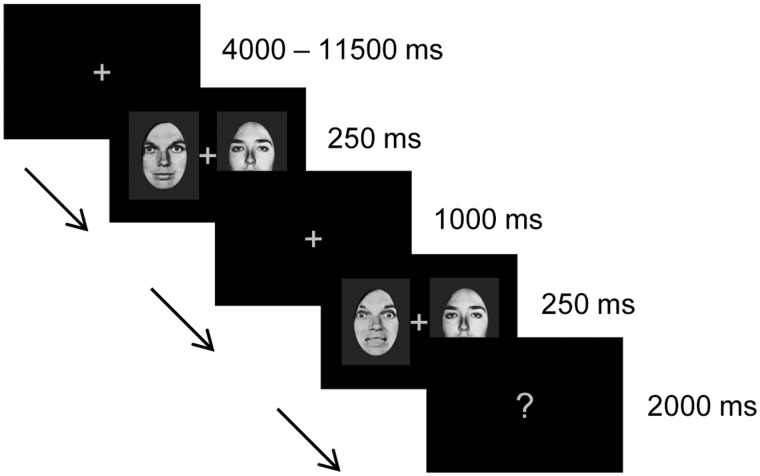
Example face trial. On each trial, two pictures of houses or faces were presented for 250 ms either side of a fixation cross. After a 1000 ms gap, with only the fixation cross remaining, a second image pair of the same stimulus class was presented for 250 ms. Participants’ task, upon subsequent presentation of a question mark, was to indicate by key-press whether there had been a change in the stimulus on the left (right index finger), in the stimulus on the right (right middle finger) or in neither stimulus (right ring finger). The trial shown here is a large ‘neutral to fear’ expression change trial where the face on the right remains the same pre and post interval. The face on the left retains the same identity but changes in expression from neutral to predominantly fearful (40% neutral, 60% fearful). The other conditions of primary interest comprised small expression changes (neutral to 60% neutral, 40% fearful) and small and large identity changes (see Materials and Methods).

All stimuli were back-projected onto a translucent screen positioned in the bore of the magnet, visible via an angled mirror placed above the participant’s head. On each trial, two images different from each other but belonging to the same stimulus category (either faces or houses) were presented on each side of a fixation cross against a black background, for 250 ms (Figure 1). Following a 1000 ms interval, during which the fixation cross was shown, a second pair of stimuli from the same category was presented in the same positions for 250 ms. A question mark at fixation then indicated that participants had 2000 ms to respond with their right hand, pressing the button under the ring finger if the two stimulus pairs were identical (33% of trials), the index finger if there was a change in the left image (33% of trials) or the middle finger if there was a change in the right image (33% of trials). Changes only occurred in one, never both images, and either involved a small or big change in identity or expression for faces or a change to a different house for houses. Participants were instructed to maintain fixation centrally throughout.

Data were acquired in five imaging ‘runs’ comprising 90 trials each, with 72 face trials (80%) and 18 house trials (20%) presented in a fixed pseudorandom order with the constraint that there was no more than three consecutive trials of the same condition. For both house and face trials, there was an equal number of no change, change to the image on the left and change to the image on the right trials. House trials were included to have an index of general change detection ability independent of the dimensions of interest (detection of changes in face identity or face expression).

The four main face conditions of interest were (i) ‘small expression’ change (neutral to 40% fear expression), (ii) ‘large expression’ change (neutral to 60% fear expression), (iii) ‘small identity’ change (100% identity A to 80% identity B, 20% identity A) and (iv) ‘large identity’ change. (100% identity A to 100% identity B). In order to prevent participants adopting a strategy of simply monitoring for fear expressions in the second pair (biasing performance on the expression trials), we added additional conditions where the first pair comprised a neutral face and either a mildly (40%) or strongly (60%) fearful face with the second pair being such that there was either no change, an expression change (the mildly or strongly fearful face being replaced with a neutral one) or an identity change (the mildly or strongly fearful face being replaced with the same level of expression from another identity). Here again, the number of no change and change on left and change on right trials was balanced to avoid response bias.

Across conditions, in order to avoid changes being detectable merely on the basis of any alteration in low or high level visual features, the second pair was 10% smaller than the first pair. Trials were separated by an inter-trial interval randomly jittered using an exponential function with a minimum of 4 s and a mean of 5.5 s. Before the beginning of the experiment, participants completed one practice block consisting of 30 trials, with the same proportion of trial types as in the main experiment, to familiarize them with the task.

### Image acquisition

Blood oxygenation level dependent (BOLD) contrast functional images were acquired with echo-planar T2*-weighted imaging (EPI) using a Siemens Tim Trio 3T MR system with a 12 channel head coil. Each image volume consisted of 48 interleaved 2 mm thick slices (interslice gap, 0.5 mm; inplane resolution, 3 × 3 mm; matrix size, 64 × 64; repetition time, 3 s; echo time, 30 ms; flip angle, 90°; bandwidth, 2232 Hz). Slice acquisition was transverse oblique, angled to avoid the eyes as far as possible while maintaining coverage of ventral temporal cortex. Data were acquired in five scanning runs of approximately 8 min. The first five volumes of each run were discarded to allow for T1 equilibration effects. T1-weighted structural images were acquired at a resolution of 1 × 1 × 1 mm.

### fMRI preprocessing

Data were analyzed using statistical parametric mapping SPM5 software (http://www.fil.ion.ucl.ac.uk/spm/). After conversion from DICOM to NIfTI format, diagnostics were run on the time series for each imaging run. Following an approach similar to that adopted by Power *et al.* (2012), see also Carp (2013), bad volumes (those with unusually high changes in mean whole brain signal intensity) were replaced by the average of the volumes on either side. These volumes were identified using the SPM timeseries diagnostic tool tsdiffana.m. Among other indices, this calculates the mean square difference of voxel-wise signal intensities, averaged across the whole volume, between each volume (*n*) and the previous volume (*n* − 1) and divides this by the mean signal across the whole volume averaged over the whole timeseries. Volumes (both *n* and *n* − 1) were rejected using an absolute cutoff (the recommended default of 10) as this handled differences between participants in the noisiness of data better than a within-participant percentile cut off. In line with findings by Power *et al.* (2012), bad volumes tended to correspond to those with notable spikes in movement. For each pair of volumes replaced, a ‘bad scan’ regressor of no interest that coded these volumes as 1 and all other volumes as 0 was created to model out the replaced volumes in the final analysis.

Subsequent to this initial data-cleaning step, slice timing correction was conducted, followed by image realignment (correcting for head movement) and normalization of each participant’s EPI data to the Montreal Neurological Institute (MNI)/ ICBM template. The latter was achieved by aligning the subject’s T1-weighted structural scan to their EPI data, then transforming the T1 into standard (MNI) space using SPM5’s combined segmentation and normalization procedure (Ashburner and Friston, 2005) and applying the same transformation to the echo-planar images. The echo-planar images were resampled to 2 mm isotropic voxels. A high-pass filter of 128 s was used to remove low-frequency noise.

### fMRI data analysis

At the single-subject level, trials were modeled with delta functions yoked to the presentation onset of the second pair of stimuli convolved with the canonical hemodynamic response function to form regressors. For each of the conditions described above, separate regressors were made for when the participant responded correctly (hits) or incorrectly (misses). The one exception was that ‘error’ trials for face no-change conditions (those with either two neutral or one neutral, one 40% fear face or one neutral, one 60% fear face) were collapsed due to the small number of error trials in each of these conditions. On ‘change’ trials, the most frequent error response was ‘no change’—there were very few instances where participants indicated a change occurred on the opposite side to that where a change actually took place (mean error rates ranged from 2% to 7% across conditions) or failed to respond (mean error rates ranged from 0.1 to 0.6% across conditions). Hence, we collapsed all error trials for each change condition under ‘misses’. Motion parameters were included in the design matrix as covariates of no interest, in addition to regressors modeling out ‘bad volumes’ that had been replaced by the average of adjacent scans during preprocessing. Beta estimates were calculated separately for each condition of interest and each run. Estimates were then averaged across runs. Given that the number of hits and misses in each condition varied across runs for each subject, we calculated a weighted mean of run-specific beta estimates for each condition in order to give higher weighting to beta estimates from runs that included a large number of trials in the condition in question and therefore had least noisy beta estimates. Specifically, the weight for each run’s beta estimate for a given condition was calculated based on [number of trials for condition X in run Y]/[number of trials for condition X across all runs].

The MarsBar ROI toolbox (http://marsbar.sourceforge.net) was used to extract mean activity (across voxels) associated with each condition of interest from our a priori regions of interest (ROIs). This was conducted using normalized but non-smoothed data. For the amygdala, we used bilateral ROIs defined by the Montreal Neurological Institute Automated Anatomical Labeling template (Tzourio-Mazoyer *et al.*, 2002). For bilateral ventrolateral prefrontal cortex (VLPFC) and fusiform face area (FFA) we used regions functionally defined in previous studies on attentional control by our group. These comprised 8-mm radius spheres centered on the following *x*, *y*, *z* coordinates (in MNI space): ± 38, 20, 0 (VLPFC), 42,−52, −20 (right FFA), −40, −50, −18 (left FFA), Bishop *et al.* (2004a, b). We have now used these ROIs across many studies, the advantage of this being clarity regarding the a-priori (as opposed to post hoc) definition of these regions. We also examined activity in bilateral dorsolateral prefrontal cortex (DLPFC), but do not report this here as it did not vary significantly for ‘hit’ *vs* ‘miss’ trials for any of the conditions of interest.

A two-way ANOVA was used to examine how activation of each of these ROIs varied as a function of change detection (hit, miss) and change type (expression, identity). It is of note that there were no significant difference in the number of hit and miss trials for identity *vs* expression trials, *F*(1,21) = 2.645, *P* = 0.119. We initially collapsed across change size to increase statistical power. Additional analyses using paired t-tests were subsequently conducted to explore whether effects identified in this main analysis held for both small and large size changes within the dimension (expression, identity) of interest. Results from these t-tests are reported one-tailed unless specified otherwise as we were testing for whether activity was greater for hit than for miss trials.

## Results

### Behavioral results

Performance was significantly above chance in all conditions (one sample *t*-tests against 33%, ts(21) > 2.2, *P*s < 0.05, two-tailed). An initial two-way analysis of variance (ANOVA) was used to determine if there was any effect of side (left or right) on which a change occurred or interaction of side x condition. No effect of side were observed (*P*s > 0.1). We hence collapsed across side of change for all further analyses. Performance accuracy (mean and standard deviation) for each condition of interest is given in Table 1.

**Table 1.** Performance in the main conditions of interest

		Partial correlations for performance (%hits) in key face conditions, controlling for performance (%hits) on house change trials				
		ID small	ID large	Exp Small	Exp Large	House
Full correlations for performance (%hits) in key conditions	ID small		0.45*	0.17	0.09	-
	ID large	0.83**		−0.05	0.15	-
	Exp small	0.54*	0.42*		0.70**	-
	Exp large	0.63*	0.63*	0.8**		-
	House	0.86**	0.82**	0.54*	0.7**	
% Mean performance ± SD		42 ± 18^a^	54 ± 17^b^	40 ± 14^a^	66 ± 18^c^	71 ± 11^c^
% Mean performance ± SD collapsed across change size		48 ± 16^d^		53 ± 15^d^		-

Cross-participant correlations between performance (% hits) in the main conditions are presented. Full correlations are given beneath the diagonal, partial correlations (controlling for performance on house change trials) above the diagonal. The full correlations reveal that, across participants, performance was strongly correlated across the four main conditions, small identity changes (ID Small), large identity changes (ID large), small neutral to fear expression changes (Exp Small), and large neutral to fear expression changes (Exp Large), as expected. A partial correlation analysis was also conducted, regressing out performance on house change trials to remove general influences on change detection performance (e.g. between participant differences in processing speed, alertness or motivation). Residual performance scores were strongly correlated within but not across change types (identity, expression), *P < 0.05, **P < 0.001. Mean performance ± standard deviation for each condition is given at the bottom of the table. Here, within each row, different subscripts indicate performance differs between conditions at *P* < 0.05. Full details of each condition of interest is provided in the Materials and Methods.

**Fig. 2.**
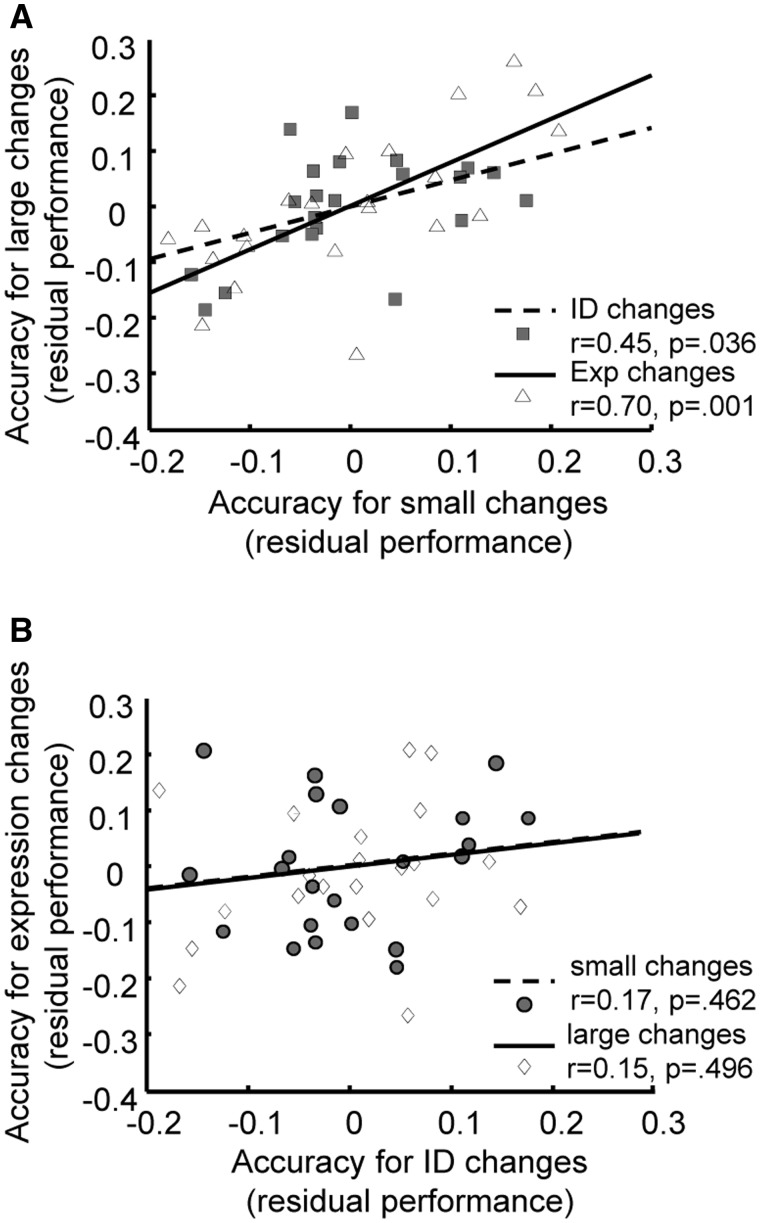
Correlations between residual performance scores, after regressing out performance on house change trials. Participants performed consistently across small and large change trials *within* each type of change (ID = identity or Exp = expression) (A) but showed far less consistency of performance within each size of change, *across* change type(B). This suggests the presence of distinct factors influencing identity *vs* expression change detection performance, across participants. (See also Table 1 for corresponding partial correlation analyses. Note d.f. for correlating residuals are 1 higher than for the partial correlations.)

In order to investigate the relative independence, across participants, in the ability to detect changes in facial expression from the ability to detect changes in identity, we examined performance in our four key conditions (same identity with a small neutral to fearful expression change; same identity with a large neutral to fearful expression change; same neutral expression with a small identity change; same neutral expression with a large identity change), with and without controlling for performance on house change trials. Full correlation analyses showed that performance was significantly correlated across all four conditions, *r*s(20)>0.4, *P*s 0.05, two-tailed, Table 1, as expected from factor analytic studies arguing for a general cognitive ability factor (‘g’) which impacts performance across tasks (Spearman, 1904; Spearman and Jones, 1950). However, partial correlation analyses controlling for performance on house change trials (and hence washing out ‘g’ effects as well as those linked to between participant differences in alertness or motivation) revealed that performance levels were correlated across difficulty levels within change type (identity or expression), *r*(19) = 0.45, *P* = 0.041 for identity changes, *r*(19) = 0.70, *P* < 0.001 for expression changes, Table 1, Figure 2A, but that there were no significant correlations between identity and expression change detection, even within the same level of difficulty, *r*s(19) < 0.2, *P*s > 0.4, Table 1, Figure 2B. This is consistent with there being between-participant differences in the ability to detect changes in facial expressions, which hold across condition difficulty levels, that are distinct from a separate dimension of individual differences in ability to detect changes in facial identities, which also holds across difficulty levels. This provides some initial support for the proposal that distinct mechanisms might facilitate detection of changes in facial expression *vs* identity.

We note that participants were primarily asked to prioritize accuracy, hence we do not report analysis of reaction time data. Further, as participants’ task was to determine whether any change occurred on the left or the right side, we can not differentiate false alarms for identity *vs* expression change trials or provide receiver operating characteristic (ROC) curves for the different conditions.

### fMRI results

#### FFA activity and detection of changes in both facial expression and identity

**Fig. 3.**
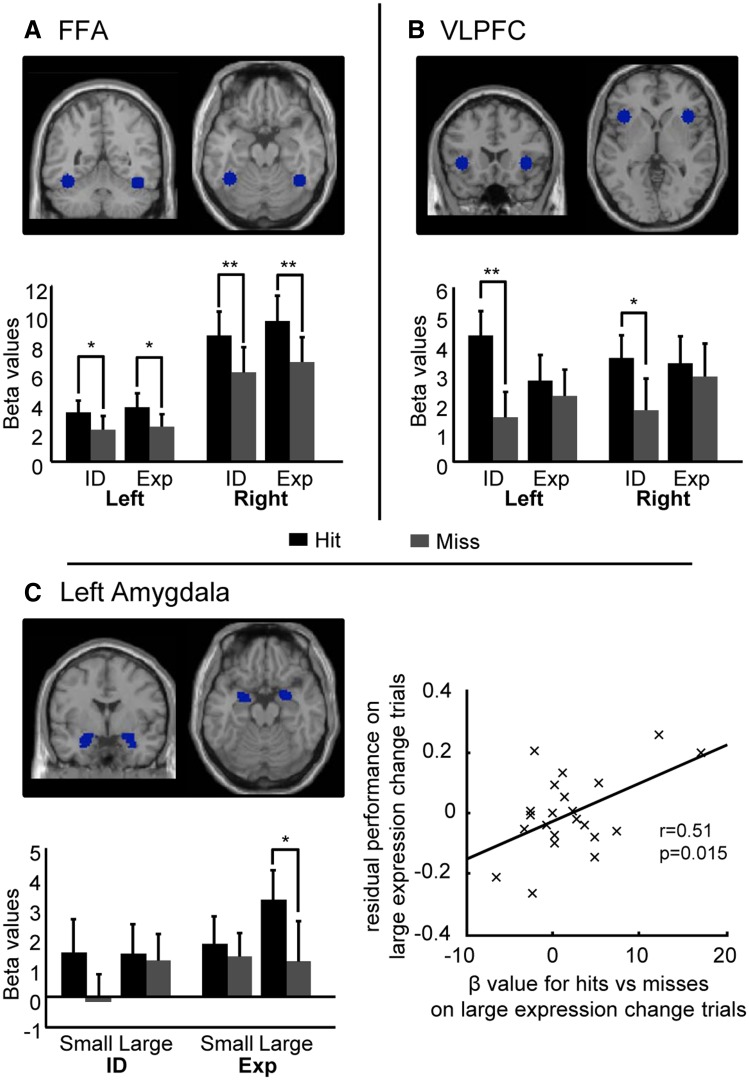
Regional activation for successfully detected *vs* missed changes on facial identity (ID) and facial expression (Exp) change trials. (A) Activity in both left and right FFA (ROIs shown in upper section of panel) was significantly greater for hits compared to misses for both identity and expression change trials. (B) VLPFC activity was greater for hits than misses for identity change trials but not for expression change trials. (C) Left amygdala activity was greater for hits *vs* misses for large expression changes (trials where expression changed from neutral to 60% fearful). Across participants, the magnitude of left amygdala activity associated with this contrast was positively correlated with performance on these large expression change trials (controlling for general change detection ability as indexed by performance on house change trials). *P < 0.05, **P < 0.005. Beta values are in arbitrary units.

We predicted that increased FFA activity would be observed for detected *vs* missed changes in both facial identity and expression, in line with this region playing a role in perception of changes in face stimuli (Fox *et al.*, 2009; Xu and Biederman, 2010). A two-way ANOVA with change detection (hits, miss) and change type (expression, identity) as factors revealed a significant main effect of change detection for both left and right FFA, F(1,21) = 9.660, *P* = 0.005, *F*(1,21) = 25.847, *P* < 0.001, respectively. There was no significant interaction between change type and detection, left FFA: *F*(1,21) = 0.036, *P* = 0.852, right FFA: *F*(1,21) = 0.072, *P* = 0.791, and separate comparison of activity to hit *vs* miss trials within each condition showed elevated bilateral FFA activity for detected (*vs* missed) changes in both identity and expression, identity change: left FFA, *t*(21) = 1.903, *P* = 0.035, right FFA, t(21) = 3.049, *P* = 0.003; expression change: left FFA, *t*(21) = 2.315, *P* = 0.015, right FFA, *t*(21) = 3.063, *P* = 0.003, Figure 3A. The effects reported here were not modulated by the size of change for either identity or expression trials, left and right FFA, *F*s < 1, *P*s > 0.4. Finally, there was also a main effect of change type in right FFA, F(1,21) = 5.321, *P* = 0.031. This reflected increased activation of right FFA in response to changes in expression compared to changes in identity when collapsing across hits and misses, *t*(21) = 2.307, *P* = 0.03, two-tailed, in line with previous findings of augmented FFA activation in response to fearful faces independent of modulatory effects of attentional (Vuilleumier *et al.*, 2001).

#### VLPFC activity and detection of changes in facial identity

A two-way ANOVA with change detection (hits, miss) and change type (expression, identity) as factors showed a significant main effect of change detection, *F*(1,21) = 6.848, *P* = 0.016, and a significant interaction between change type and change detection, *F*(1,21) = 6.404, *P* = 0.019, in left VLPFC. To break this interaction down, we separately compared activity for hits *vs* misses for identity and for expression trials. This revealed that for identity change trials, left VLPFC activity was higher on trials where changes were successfully detected than when they were missed, *t*(21) = 4.195, *P* < 0.001. In contrast, this pattern was not found for changes in expression, *t*(21) = 0.593, *P* = 0.280, Figure 3B. Planned comparisons confirmed that this increased activation for hits *vs* misses was significant for both small and large changes in identity, *t*(21) = 2.366, *P* = 0.014, *t*(21) = 4.142, *P* < 0.001, respectively, but not for small or large changes in expression, *t*(21) = 1.334, *P* = 0.098, *t*(21) = −0.194, *P* = 0.576, respectively. Activity to hits *vs* misses did not differ significantly between large and small identity change trials, *t*(21) = −0.198, *P* = 0.84, contrary to any ‘task difficulty’ interpretation of this activation.

In right VLPFC, we found no significant main effects of change type or change detection or interaction of change type by change detection, *P*s > 0.1. Planned *t*-tests comparing hits and misses for each condition did however suggest a similar but weaker pattern of activation to that observed in left VLPFC: activity was increased for hits *vs* misses for identity trials, *t*(21) = 1.875, *P* = 0.038, but not for expression trials, *t*(21) = 0.383, *P* = 0.353, Figure 3B.

##### Amygdala activity and detection of changes in facial expression

Left amygdala activity was greater for trials where changes were detected than missed, across change types, *F*(1,21) = 4.698, *P* = 0.042. Although the interaction of change detection by change type was not significant, planned *t*-tests revealed that left amygdala activity was higher in response to correctly detected changes for expression trials alone; expression change trials: *t*(21) = 1.970, *P* = 0.031, identity change trials: *t*(21) = 1.058, *P* = 0.15. Consideration of large and small changes separately showed that this effect was only significant for large changes in expression, *t*(21) = 1.825, *P* = 0.041, Figure 3C. Across participants, left amygdala activity for detected *vs* missed large expression changes was positively correlated with change detection performance on large expression change trials, *r*(20) = 0.51, *P* = 0.015, two-tailed, Figure 3C. Here, residual performance scores were used, after regressing out performance for house change trials to control for generic aspects of change detection performance, as described earlier (see Behavioral results, Table 1 and Figure 2). No parallel relationship between amygdala activity for hits *vs* misses and change detection performance was observed for small expression change trials (*P* > 0.1) or for small or large identity change trials, (*P*s > 0.5.). No significant effects of interest were observed within the right amygdala.

## Discussion

In line with our first hypothesis, successful detection of changes in face stimuli was associated with heightened FFA activity regardless of whether facial expression or facial identity changed. This finding is consistent with occipital–temporal cortical regions playing a role in perception of changes in their preferred stimulus category. This result replicates previous findings for facial identity (Beck *et al.*, 2001) and also indicates FFA responsivity to changes in facial expression. Initially it was thought that the FFA mainly encoded facial identity and not facial expression (Haxby *et al.*, 2000). However, there is now increasing evidence that the FFA is also sensitive to changes in facial expression (Fox *et al.*, 2009; Xu and Biederman, 2010; Bishop *et al.*, 2015). Further, findings from adaptation studies contrasting changes that either do or do not cross perceptual categorization boundaries (the point where a face is perceived as being a different identity or showing a different expression) suggest that FFA adaptation tracks perceived as opposed to physical differences in facial stimuli (Fox *et al.*, 2009). In the light of this, our current results are consistent with an increase in FFA activity to hit *vs* miss face ‘change’ trials indexing the perception of a change in the facial stimuli observed.

In line with our second hypothesis, increased VLPFC activity was only associated with correct, *vs* failed, detection of changes in facial identity and not correct, *vs* failed, detection of neutral to fearful changes in expression. Here, our hypothesis was informed by the proposal that allocation of top-down attention to monitoring for changes in task-relevant stimuli would facilitate detection of low saliency changes such as those in facial identity to a greater extent than detection of high saliency changes, such as those in expression. The latter were predicted, instead, to be primarily determined by bottom-up mechanisms enabling attentional capture by high saliency changes. In support of this proposal, VLPFC has previously been implicated in facilitating the detection of task-relevant events, of low but not high perceptual salience, when they occur at un-cued spatial locations (Indovina and Macaluso, 2007; Chica *et al.*, 2013). More generally VLPFC is thought to be part of a circuit that facilitates top-down attentional control, in particular the allocation of attentional resources to task relevant stimulus features (Thompson and Duncan, 2009). Further, in the case of change detection, trial to trial variations in frontal activity prior to change occurrence have been shown to covary with success in detecting changes in facial identity; pre-change activity being greater on hit than on miss trials (Pourtois *et al.*, 2006). This is in line with intrinsic fluctuations in top-down attention influencing performance. In this context, our current findings provide additional support for the proposal that VLPFC activity is linked to successful detection of low saliency, but task-relevant, events—specifically the detection of low saliency changes in task stimuli (i.e. in facial identity) but not high saliency changes (i.e. from neutral to fearful expressions).

Here, it is worth noting that while Beck *et al.* (2001) primarily reported change detection activity in DLPFC, a number of subsequent reports have linked activity in VLPFC to successful change detection, detection of task-relevant cues, and rejection of close foils in target detection paradigms (Pessoa and Ungerleider, 2004; Hampshire *et al.*, 2008, 2010). We also note that other studies have argued for a generic role for frontal regions in visual awareness (Rees, 2007). Within the current study, a general correlate of change awareness would be expected to be indexed by activity to hit trials *vs* miss trials, regardless of change type. It hence seems unlikely that differential engagement of VLPFC to hit *vs* miss trials for identity *vs* expression changes purely reflects a role of this region in change awareness.

We further predicted that while frontal activity would not discriminate detected *vs* non-detected changes from neutral to fearful expressions, these trials would instead be discriminated by differential amygdala activation. This follows findings from across a range of paradigms (backward masking, binocular disparity, attentional blink tasks) that amygdala activation is observed for extremely briefly presented fearful expressions (Whalen *et al.*, 1998, 2004) and associated with the capture of attentional resources by such stimuli (Amting *et al.*, 2010; Schwabe *et al.*, 2011; Pourtois *et al.*, 2013). In line with this, we observed increased left amygdala activity for detected *vs* missed changes when the expression of one of the two faces changed from neutral to predominantly fearful (60% fearful, 40% neutral). The failure to find this for small expression changes is perhaps not unexpected, given that many reports have suggested that expression perception follows more of a step function than a linear function (Calder *et al.*, 1996), and as such an expression change from 100% neutral to 60% neutral, 40% fear may not have been sufficient to cross the perceptual boundary and recruit the amygdala to reorient attention towards the face in question. Collapsing across small and large change trials, elevated amygdala activity was significantly associated with successful detection of neutral to fearful changes in expression, but not with successful detection of changes in identity. The interaction of change type by detection success (hits *vs* misses) did not however reach significance. The power to detect this was probably weakened by inclusion of the small neutral to fear changes in expression. Future work aimed at replicating this finding that focuses solely on changes that cross the perceptual boundary will be of value.

The finding that, within-subjects, there was an increase in amygdala activation for detected as opposed to missed large neutral to fear expression changes need not necessarily indicate engagement of a mechanism that facilitates detection of such changes, as proposed here. Alternatively, this activation could reflect awareness of such changes, or post change-detection recruitment of the amygdala to engage flight or fight responses (see Pessoa *et al.*, 2006 for a discussion of this issue in the context of backward masking). However, the finding that, across participants, those showing greater amygdala activity for hit *vs* miss trials in the large neutral to fear expression change condition also achieved better performance on these trials suggests that this amygdala activation is more likely to reflect a mechanism that supports change detection as opposed to one that merely reflects some sequelae of it. If this were not the case, regardless of how many neutral to fear change trials were correctly detected, we would expect to observe similarly elevated activity to hits *vs* misses across individuals.

In summary, our findings suggest that ‘top-down’ frontal and ‘bottom-up’ amygdala attentional mechanisms may differentially facilitate detection of changes in facial identity *vs* facial expression, respectively, with bottom up amygdala-driven capture of attention enabling detection of threat relevant changes in expression and overriding the influence of trial to trial variations in VLPFC activity. Activity in this latter region was linked to successful performance on identity change trials, in line with VLPFC facilitating task-oriented attention, and in particular the detection of un-cued, task-relevant, but low saliency changes. In future work it will be of interest to determine whether the link between amygdala activation and change detection performance is specific to threat-relevant changes in expression or is observed more widely, for example in the case of changes towards positive expressions, as well for changes that are highly salient as a result of other stimulus properties.

*Conflict of interest*. None declared.

## Funding

This study was support by Medical Research Council grant G120/919 and NIMH grant RO1MH091848.
